# A novel *Schistosoma japonicum* endonuclease homologous to DNase II

**DOI:** 10.1186/s12864-015-1319-5

**Published:** 2015-02-25

**Authors:** Nan Hou, Xianyu Piao, Pengfei Cai, Chuang Wu, Shuai Liu, Yan Xiao, Qijun Chen

**Affiliations:** MOH Key Laboratory of Systems Biology of Pathogens, Institute of Pathogen Biology, Chinese Academy of Medical Sciences & Peking Union Medical College, Dong Dan San Tiao 9, Beijing, People’s Republic of China; Key Laboratory of Zoonosis, Ministry of Education, Institute of Zoonosis, Jilin University, Xi An Da Lu 5333, Changchun, People’s Republic of China

**Keywords:** *Schistosoma japonicum*, Nuclease, DNase II homologue, Host–parasite interaction

## Abstract

**Background:**

Recent advances in studies of the *Schistosoma japonicum* genome have opened new avenues for the elucidation of parasite biology and the identification of novel targets for vaccines, drug development and early diagnostic tools.

**Results:**

In this study, we surveyed the *S. japonicum* genome database for genes encoding nucleases. A total of 130 nucleases of 3 classes were found. Transcriptional analysis of these genes using a genomic DNA microarray revealed that the majority of the nucleases were differentially expressed in parasites of different developmental stages or different genders, whereas no obvious transcriptional variation was detected in parasites from different hosts. Further analysis of the putative DNases of *S. japonicum* revealed a novel DNase II homologue (Sjda) that contained a highly conserved catalytic domain. A recombinant Sjda-GST protein efficiently hydrolysed genomic DNA in the absence of divalent iron. Western-blot and immunofluorescence assays showed that Sjda was mainly expressed on the teguments of female adult parasites and induced early humoral immune responses in infected mice.

**Conclusions:**

A novel DNase II homologue, Sjda, was identified in *S. japonicum*. Sjda was mainly distributed on the teguments of adult female parasites and possessed a typical divalent iron-independent DNA catalytic activity. This protein may play an important role in the host–parasite interaction.

**Electronic supplementary material:**

The online version of this article (doi:10.1186/s12864-015-1319-5) contains supplementary material, which is available to authorized users.

## Background

Schistosomiasis is one of the most serious parasitic diseases, infecting over 200 million people in 76 tropical and subtropical countries [1]. The pathogenesis of schistosomiasis is mainly caused by egg-induced granuloma formation and subsequent fibrosis. However, tools for the early diagnosis and abrogation of the pathophysiological effects of the parasite, especially the eggs, are still lacking. Treatment of schistosomiasis has relied on a single drug, praziquantel; however, strains of *S. mansoni* that are resistant to praziquantel have emerged [2]. Understanding the parasite biology and the mechanism of the host–parasite interaction are critical for the development of an effective vaccine and anti-parasite drugs [3], which are urgently needed for schistosomiasis control.

Recent advances in research on the *S. japonicum* genome, transcriptome, and proteome have provided information that contributes to the understanding of parasite biology and the host–parasite interplay [4-7]. Nucleases, including DNases, RNases, topoisomerases, recombinases, ribozymes, and RNA splicing enzymes, have diverse functions ranging from DNA replication, recombination and repair and RNA maturation and processing to nutrient regeneration and cell death in various species [8]. Recent evidence has highlighted a novel role of nucleases, especially DNases, in pathogen evasion of host defence mechanisms. Nucleases of bacteria, such as *Streptococcus* [9-11], *Staphylococcus aureus* [12] and *Aeromonas hydrophila* [13], have been suggested to act as virulence factors in resistance to host neutrophil extracellular traps (NET), while members of the DNase II family in the roundworm *Trichinella spiralis* have been found to be secreted into circulation to counteract host innate immune responses [14]. Thus, disrupting the functions of vital nucleases will not only hinder the homeostasis and development of parasites but also be beneficial to the host immune system and allow parasite control. However, no nucleases have been identified and characterised in the *Schistosoma* genus to date, except for a dicer enzyme in *S. mansoni* [15]. In this study, we first surveyed the *S. japonicum* genome for genes encoding potential nucleases and then analysed the transcription of these genes during different developmental stages using a DNA microarray. Numerous genes encoding potentially important nucleases were identified, and a novel DNase II predominantly expressed during the mature parasite stage was systematically characterised.

## Results

### Identification and characterisation of putative nuclease sequences of *S. japonicum*

Using known nucleases from *Brugia malayi*, *Caenorhabditis briggsae*, *Caenorhabditis elegans*, *Hydra vulgaris*, *Nematostella vectensis*, *Schistosoma mansoni* and *Trichoplax adhaerens* as bait sequences, 238 homologous proteins encoded in the *S. japonicum* genome were identified. Of these, 130 proteins were found to contain domains with potential nuclease activities. SignalP4.0 was also used to predict signal peptides. Potential proteins with a D score of greater than 0.45 were considered to have an N-terminal signal peptide sequence. Among the 130 nucleases, 12 were predicted to have signal peptide sequences. The proteins were also characterised according to their substrates, enzymatic properties, and divalent cation dependencies. The catalytic activities of 24 nucleases were potentially divalent cation-dependent (Table 1). The potential nucleases were grouped into three classes as follows: 4.6% were classified as DNases, 60.8% as RNases, and the remainder (34.6%) possessed the potential for hydrolysing both DNA and RNA (DNase/RNase).

**Table 1:** **The characteristics of the putative nuclease sequences of** ***S. japonicum***

**Nuclease class**	**Num. nuclease sequences**	**Enzymatic properties of nucleases**			**Nucleases with signal sequence**	**Divalent cation-independent nucleases**
		**Exo**	**Endo**	**Exo/endo**		
DNase	6 (4.6%)	1	2	3	1	4
RNase	79 (60.8%)	20	55	4	2	6
DNase/RNase	45 (34.6%)	5	30	10	9	11
Sum	130	26	87	17	12	24

### Transcriptional analysis of genes encoding putative nucleases of *S. japonicum*

**Figure 1:**
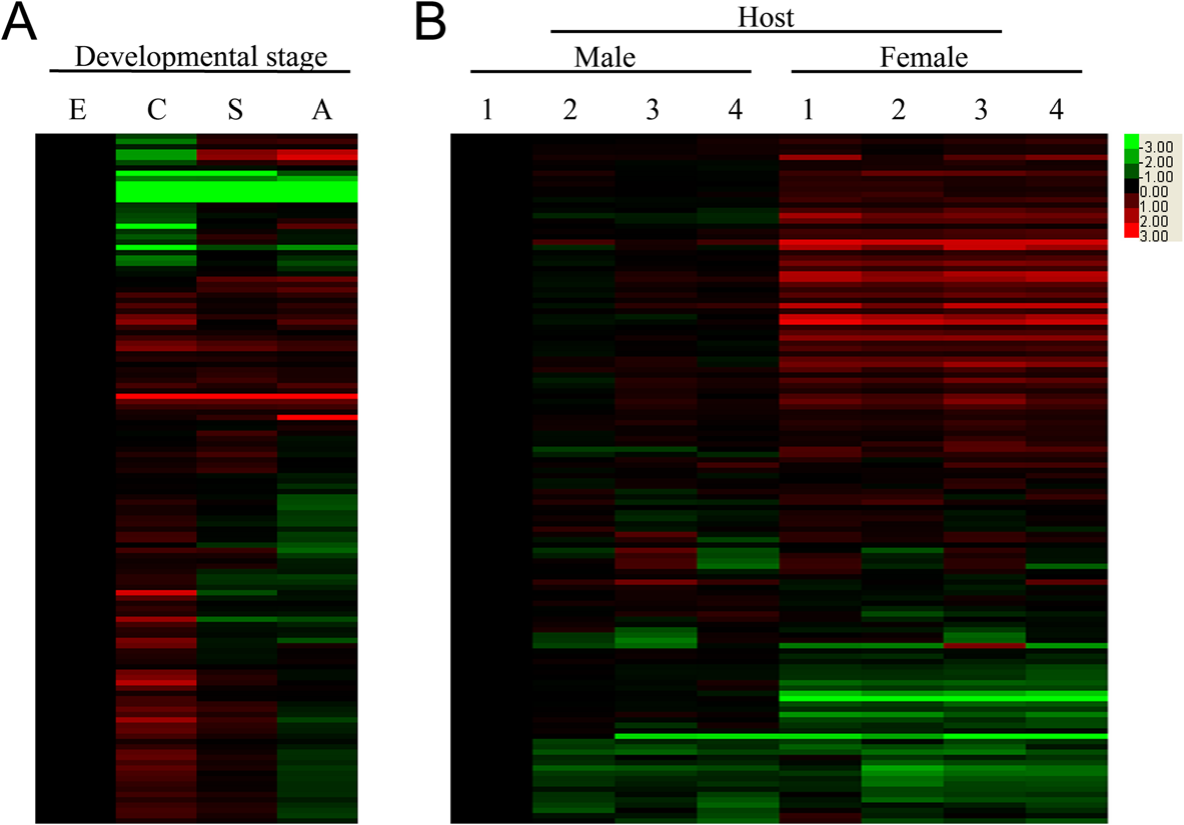
**Heat maps of transcription of all putative nucleases in** ***S. japonicum*** **parasites of different stages, genders or hosts. A.** Transcription of all putative schistosome nucleases from parasites at different developmental stages. The cercariae (C) were obtained from infected *Oncomelania hupensis*, and the eggs (E), hepatic schistosomula (S) and paired adult worms (A) were obtained from *S. japonicum*-infected BABL/c mice. **B.** Transcription of all putative nucleases from parasites of different genders and hosts. Male and female adults were obtained from *S. japonicum*-infected mammals, including BABL/c mice (1), C57BL/6 mice (2), rabbits (3) and buffaloes (4). The transcriptional data is based on a genomic DNA microarray dataset.

**Figure 2:**
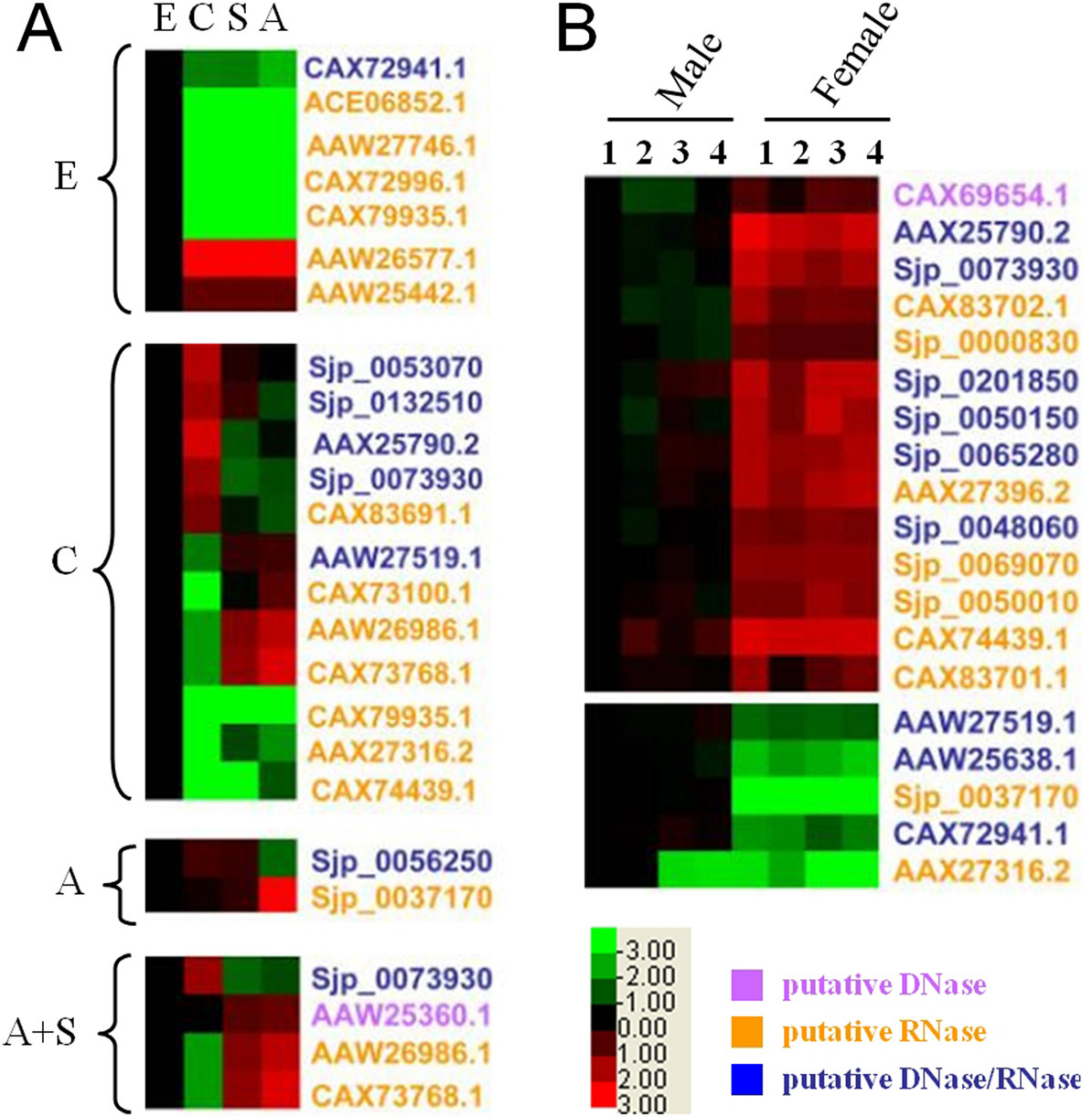
**Heat maps of nuclease genes with distinct transcription profiles from parasites of different stages or genders. A.** Genes encoding putative nucleases with distinct transcriptional patterns in parasites of different developmental stages. The parasites were obtained as described in Figure 1. **B.** Genes encoding putative nucleases with distinct transcriptional patterns in adult parasites of different genders. A ≥2-fold difference was consistent with differential expression (*p* < 0.05) throughout the life cycle or between the female and male adults.

The transcriptional characteristics of the putative nucleases of *S. japonicum* were investigated using a target-specific microarray. The expression of the nucleases greatly differed among the parasites at different developmental stages (Figure 1A) and between those of different genders (Figure 1B), whereas few differences were observed among adult worms of the same gender isolated from different hosts (Figure 1B). There were a total of 22 genes that were distinctly expressed at particular developmental stages (Figure 2). A total of 19 genes were differentially expressed in the male or female adults from various hosts, including1 DNase, 6 nucleases and 7 RNases, which were up-regulated in the female adults, and 3 nucleases and 2 RNases, which were up-regulated in the male adults (Figure 2B).

**Figure 3:**
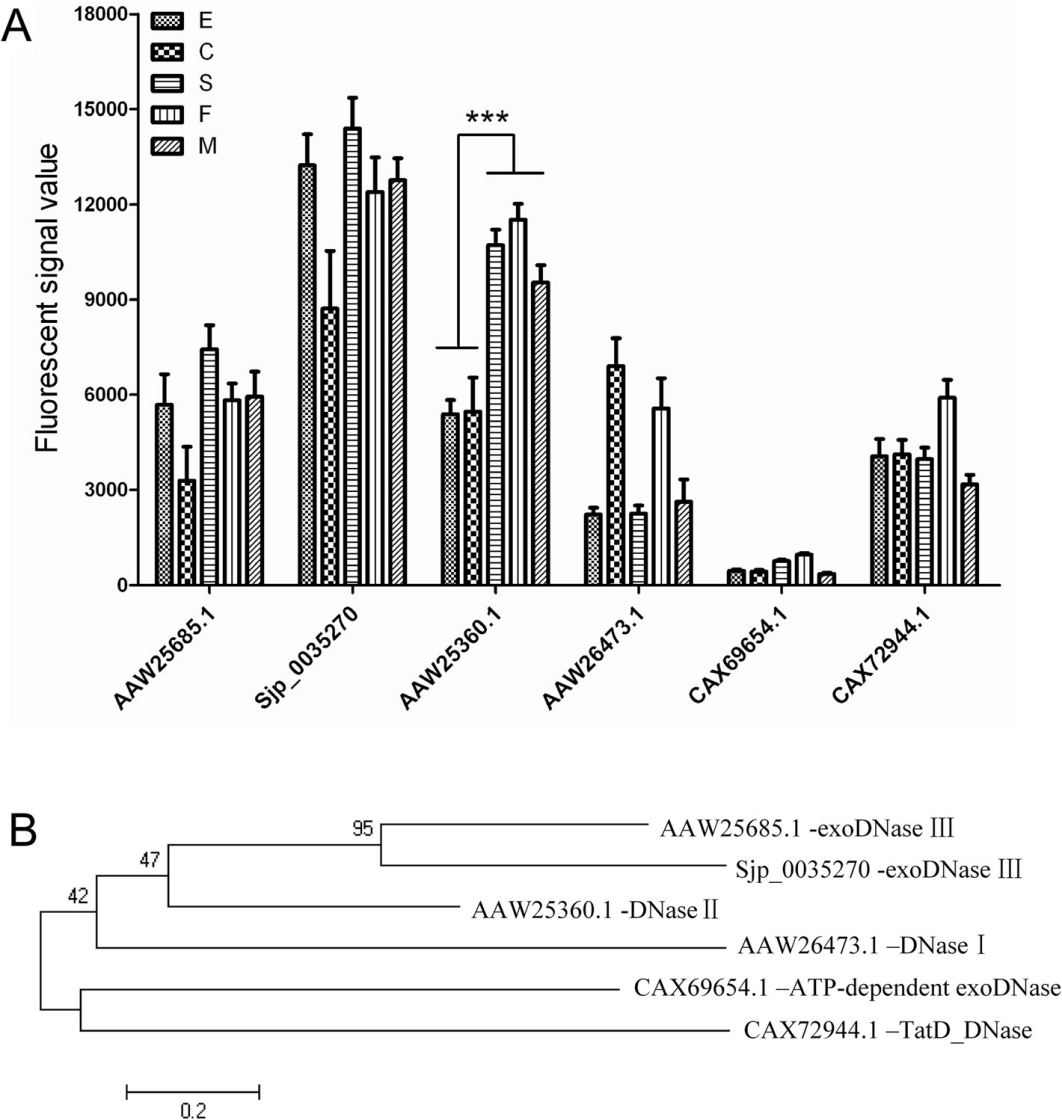
**Transcriptional characteristics and phylogenetic classification of putative schistosomal DNases. A.** Quantitative comparison of the transcriptional data of the putative DNases in parasites of different developmental stages (E: egg; C: cercariae; S: schistosomulum; M: male; and F: female) based on a DNA microarray dataset. *** indicates *p* < 0.001. **B.** Phylogenetic tree constructed with amino acid sequences of the putative DNases. A scale of 0.2 is shown below the tree. The frequency of each sequence is shown in brackets before the name.

**Figure 4:**
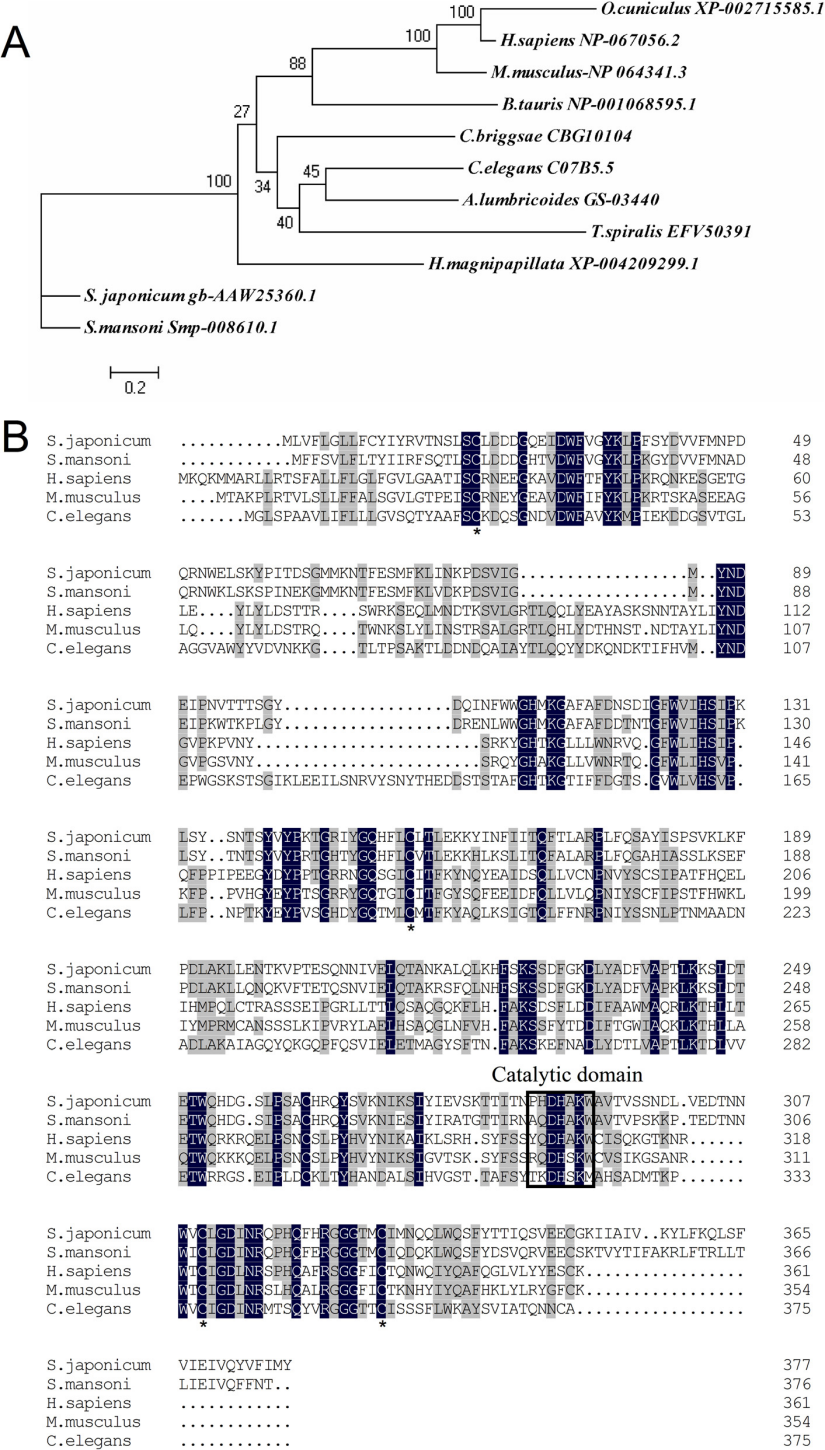
**Comparison of amino acid sequence of Sjda with DNaseII molecules from various species. A.** Phylogenetic tree constructed with amino acid sequences of Sjda and 10 DNase II molecules from other species. The protein name includes the species name and NCBI protein bank ID. A scale of 0.2 is shown below the tree. The frequency for each sequence is shown in brackets before the name. **B.** Amino acid sequence alignments of Sjda and *S. mansoni*, human, mouse and *C. elegans* DNase II homologues. The catalytic domains are enclosed inboxes. * indicates conserved cysteine residues. Residues that are present in all sequences are highlighted with dark-grey shading. Residues that are conserved in at least three sequences are highlighted in grey. The dots indicate missing residues.

The transcription of the 6 putative DNases was further analysed. Among them, only the expression of AAW25360.1 was significantly up-regulated in the schistosomula and paired adults compared with egg and cercariae (Figure 3A). These results were confirmed by QPCR (data not shown). A phylogenetic comparison showed that these DNases were clearly divided into 5 major groups, including DNase I, DNase II, exoDNase III, ATP-dependent exoDNase and Tat DNase (Figure 3B). AAW25360.1 was separate from the other DNases (Figure 3B). The similarity of AAW25360.1 with known DNase II sequences from other organisms was analysed (Figure 4A). AAW25360.1 shared 70.3%, 25.4%, 27.5%, and 25.4% identity with the DNase II proteins of *S. mansoni*, human, mouse and *C. elegans*, respectively. The full-length cDNA of AAW25360.1 contained an open reading frame of 1,134 bp encoding 378 amino acids with a molecular mass of 43,607 Daltons, and it was termed Sjda (*S. japonicum* DNase). Sjda contained conserved amino acid residues, especially in the catalytic domain, and had a composition typical of DNaseII nucleases (Figure 4B and data not shown). It also had a classical peptide sequence at the N-terminus.

### Characterisation of DNase activity of Sjda

**Figure 5:**
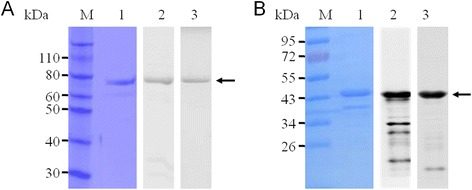
**Generation of recombinant Sjda proteins. A.** Detection of purified GST-tagged recombinant Sjda protein. The molecular weight of Sjda-GST is 69 kDa. **B.** Detection of purified His-tagged recombinant Sjda protein. The molecular weight of Sjda-His is 43.5 kDa. Lane M, protein molecular weight markers; Lane 1, purified proteins were analysed by SDS-PAGE and stained with Coomassie brilliant blue; Lane 2, Western blotting of the recombinant protein with specific mouse anti-Sjda antibodies; and Lane 3, Western blotting of the recombinant protein with anti-GST-tag mAb (A) or anti-His-tag mAb (B).

**Figure 6:**
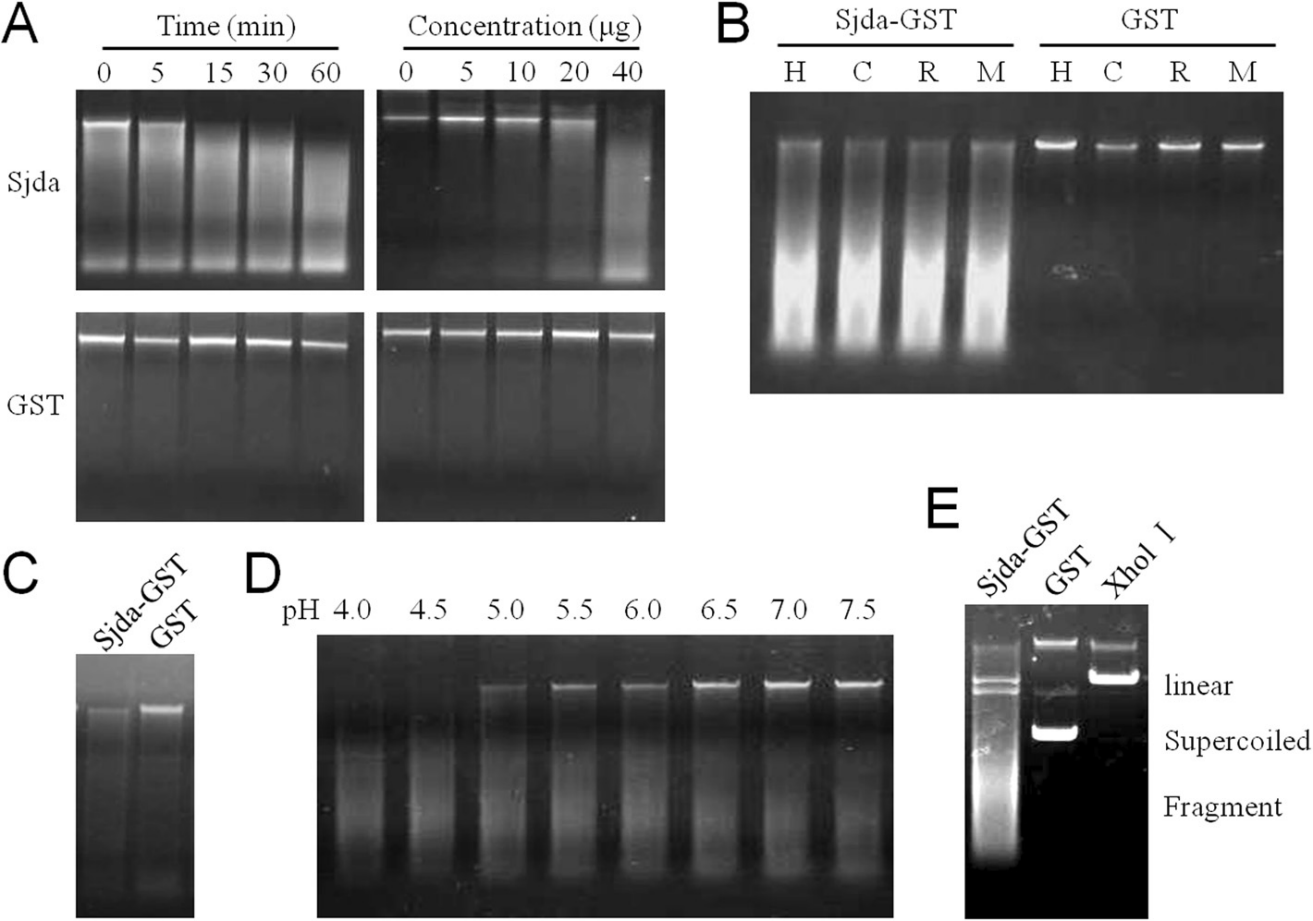
**DNase activity of the recombinant protein Sjda-GST.** Agarose gel electrophoresis was carried out to analyse the catalytic products of the DNase. **A.** The left panels show the DNA digested by 40 μg Sjda-GST or GST proteins at the indicated times. The right panels show the digested DNA according to the indicated amount of Sjda-GST or GST proteins with an incubation time of 15 min. All assays were carried out with 200 ng human genomic DNA in a final volume of 25μlPBS (pH7.0) at 37°C. **B.** The DNA from different hosts digested by Sjda-GST or GST. H, human; C, calf; R, rabbit; and M, C57BL/6 mouse. **C.** *S. japonicum* DNA digested by Sjda-GST or GST. **D.** Human DNA digested by Sjda-GST in PBS of different pH levels, ranging from 4.0 to 7.5. **E.** Supercoiled plasmid DNA digested by Sjda-GST, GST or XhlI.

To confirm the endonuclease activity of Sjda, a recombinant Sjda-GST protein and Sjda-His were generated (Figure 5A and B) and tested for DNA hydrolysis activity. Sjda-GST could efficiently digest genomic DNA from human, bovine, rabbit and murine sources, and its catalytic activity was independent of any divalent cation (Figure 6A, B). Sjda could also digest the genomic DNA of *S. japonicum* (Figure 6C). We next determined the optimum pH for its activity and found that Sjda-GST activity occurred over abroad pH range and was highly active at acidic pH levels (Figure 6D). A further assay with supercoiled plasmids as substrates showed that Sjda-GST-digested products were not only short fragments but also fragments that were slightly smaller than the linear DNA produced by XhoI (Figure 6E), indicating that it may also be able to produce nicks along DNA chains apart from double-strand digestion.

### Sjda was mainly identified on surface membranes of female adult worms

**Figure 7:**
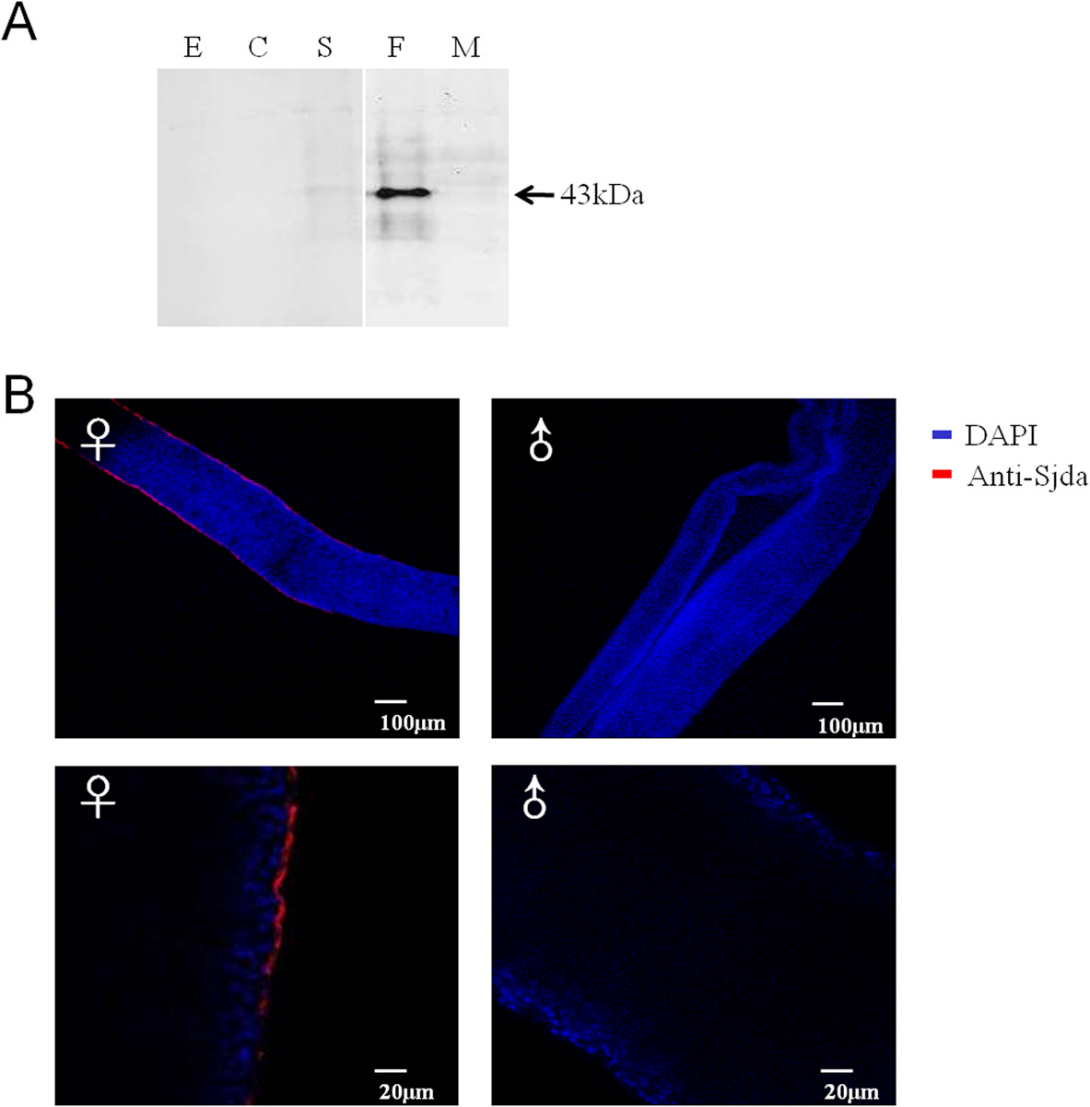
**Detection of Sjda protein in parasites of** ***S. japonicum*** **. A.** Detection of Sjda protein in parasites at different developmental stages with specific anti-Sjda antibodies by Western blotting. A specific band is present only for the proteins extracted from the female parasite. **B.** Detection of the Sjda protein on the surfaces of adult worms by immunofluorescence. A specific signal (in red colour) was observed only on the teguments of the female parasite. The lower panels depict magnifications of the upper panels. ♀, female adult; and ♂, male adult.

To confirm Sjda expression, a Westernblot assay was first carried out to detect this protein in parasites at different developmental stages. A thick protein band for Sjda was observed in the female adults, and a dim band was detected in the schistosomula, while no band was observed in the eggs, cercariae or male adults (Figure 7A). These results indicated that Sjda was mainly expressed in the female parasites. Immunofluorescence further confirmed that this protein was mainly localised to the surfaces of the female parasites (Figure 7B).

### Dynamics of Sjda-specific antibodies in infected hosts

Sjda-specific IgG levels in the sera of infected mice on days 0, 7, 14, 28, 42 post-infection were detected by Western blotting. The Sjda-His protein was specifically recognised by IgG in some infected mice as early as day 7 post-infection. This immune reactivity increased gradually over time, peaking on day 42 (Additional files 1 and 2). The serum positive rates of Sjda-specific IgG on days 0, 7, 14, 28, 42 were 0, 50 ± 14.1, 77.4 ± 8.4, 91.7 ± 11.8, and 100 ± 0%, respectively (Additional file 1).

## Discussion

Nucleases, particularly DNases, have recently been found to play novel roles in pathogen invasion in evading host defence mechanisms. For example, the M1 serotype strain of Group A *Streptococcus* (GAS) has been found to express a potent DNase that is both necessary and sufficient to promote GAS neutrophil resistance and virulence in a murine model of necrotising fasciitis [9]. The membrane-attached surface-exposed DNA-entry nuclease EndA of *Streptococcus pneumoniae* has been shown to play an important role during the establishment of invasive infections by degrading extracellular chromatin and thereby overcoming the innate immune responses in mammals [10]. *S. pyogenes* nuclease A promotes bacterial survival in whole human blood and in neutrophil killing assays [11]. The expression of nucleases by *S. aureus* has also been found to lead to increased bacterial dissemination in mice [12]. In addition to those found in bacteria, a multitude of DNase family members have been found in *T. spiralis*. These DNases are believed to enhance the degradation of DNA released from host phagocytes, which are involved in the down-regulation of host inflammatory processes [14]. In this study, we performed a genome-wide investigation to identify the nucleases of *S. japonicum* with potential functions in host-parasite interactions.

We identified 130 nucleases of 3functional classes, which all contained conserved domains of classical nucleases (Table 1 and Additional file 3). The majority of these nucleases were found to be differentially expressed in eggs, cercariae or flukes (including schistosomula and adults), but only a few genes were differentially expressed between the schistosomulum and adult stages. This finding indicated that the schistosomula and adults have similar nucleicacid metabolic pathways. Furthermore, the expression levels of the nuclease genes in adult worms of the same gender from different hosts were also similar, indicating that the parasites have evolved a capacity for adaptation to different host environments (Figures 1 and 2). Additionally, the expression levels of nucleases were greatly increased in the female parasites compared with the male parasites (Figure 1B), suggesting that they are critical components that are much more metabolically active in female compared with male parasites. However, it cannot be ruled out that some nucleases actively participate in the host-parasite interaction.

Among all 130 putative nucleases, six genes encoding putative DNases were identified in the *S. japonicum* genome. These DNases only accounted for 4.6% of all of the putative nucleases. AAW25360.1 (Sjda), with a sequence characteristic of DNase II, was the only DNase that showed significantly increased expression in the adult parasites (Figure 3A). Since the identification of human DNase II in 1998, murine [16], porcine [17] and bovine [18] DNase II genes have been sequentially identified. Other putative genes encoding proteins homologous to DNase II have recently been observed in the genomes of many species, including *Gallus gallus* (chicken), *Fugu rubripes* (puffer fish), *Xenopus laevis* (frog), *Anopheles gambiae* (mosquito), *Drosophila*, and the slime mould *Dictyostelium discoideum*, in addition to parasitic organisms, such as *Trichinella spiralis*, *Trichomonas vaginalis*, and the bacterium *Burkholderia pseudomallei* [19]. Analysis of these DNase II genes and their homologues has revealed a striking conservation of amino acid residues surrounding the catalytic site of the molecule [20]. The high conservation of amino acid residues in the catalytic domain of Sjda in the DNase II family (Figure 4B) strongly indicates that its function is similar to those of other DNase II members.

The catalytic activity of the recombinant Sjda-GST protein was divalent cation-independent, and its preference for an acidic environment led to its characterisation as an endogenous DNase II (Figure 6A-D). However, it seemed to be able to introduce nicks along one strand of the DNA, similar to human DNase I [16] (Figure 6E). Sjda was mainly up-regulated in the schistosomula and adult parasites, but not in the cercariae or eggs, suggesting that this molecule may play a role in the adaptation of parasites to the host circulation (Figure 7A). The expression of Sjda was mainly localised to the surfaces of the adult female parasites, but it was not detected in the gut, further supporting this hypothesis (Figure 7B). Interestingly, Sjda was not expressed in the male parasites (Figure 7B). Previous studies have shown that the teguments of male and female parasites are structurally different, and the surfaces of male schistosomes are coated with an increased number of sugar components compared to those of female parasites [21]. These findings indicate that male and female parasites possess different arsenals for the resistance of host immune expulsion.

Furthermore, although Sjda was primarily found on the surfaces of the female parasites, it cannot be ruled out that it is secreted by the parasite. It has been reported that *Trichinella spiralis* constantly secretes DNase II into the surrounding environment in the host and affects the host immune system [22,23]. We predicted the localisation of a signal peptide sequence with 19 amino acids to the N-terminus of Sjda. This finding strongly indicates that it is secreted by the parasite. Furthermore, the detection of Sjda-specific antibodies in the infected host serum as early as one week after infection also suggested that it was recognised by the host immune system and played a role in the host-parasite interaction.

## Conclusions

In this study, we discovered a novel DNase II homologue, Sjda, with a DNA hydrolysis function. This protein was found to be mainly expressed on the tegument of female adult parasites, and it also stimulated early humoral immune responses after infection. Sjda is likely to play an important role in the host–parasite interaction. These findings provide a novel starting point for the discovery of novel targets for drug and vaccine development against schistosomiasis.

## Methods

### Ethical statement

All procedures performed on the animals in this study were conducted according to the animal husbandry guidelines of the Chinese Academy of Medical Sciences. The human peripheral blood sample was donated by a healthy volunteer and anonymised. Written informed consent was obtained from this volunteer for the publication of this study and any accompanying images. A copy of the written consent is available for review by the Editor of this journal. All procedures performed on human samples were carried out in accordance with the tenets of the Declaration of Helsinki. This research was reviewed and approved by the Experimental Animal Committee and the Ethical Committee of the Chinese Academy of Medical Sciences.

### Parasites and animals

Parasite-infected *Oncomelania hupensis* was purchased from the Hunan Institute of Parasitic Diseases, Yueyang, China. The freshly released cercariae were harvested immediately. Special pathogen-free (SPF) C57BL/6 mice (males, 6 weeks old) and New Zealand white rabbits (both from the Vital River Laboratory Animal Technology Co. Ltd., Beijing, China) were percutaneously infected with *S. japonicum* cercariae (20 parasites per mouse or 1,000 to 1,500 per rabbit). Sera from the infected animals were collected on days 0, 7, 14, 28 and 42. Hepatic schistosomula were isolated from the animals at 2 weeks post-infection, while mixed adult worms were obtained at 6 weeks post-infection by portal perfusion and manual separation under a light microscope. Eggs were purified from the liver tissues as previously described [24]. Adult worms from infected buffaloes were provided by the Hunan Institute of Parasitic Diseases.

### Total RNA isolation and quality control

Parasites of all stages were soaked in RNAlater solution (Ambion, CA, USA) overnight and stored at −80°C. Total RNA was extracted using an RNeasy Mini Kit (QIAGEN GmbH, Hilden, Germany), and the contaminating genomic DNA was completely removed from the RNA samples with a TURBO DNA-free™Kit (Ambion). RNA quantification and quality control was conducted with a Nanodrop ND-1000 spectrophotometer (Thermo Fisher Scientific, Wilmington, DE, USA) and denaturing agarose gel electrophoresis.

### Microarray construction, hybridisation and validation

The sequences used for the design and construction of the microarray were obtained from three databases and included putative transcriptome data for *S. japonicum* (12,657 sequences) [25] and *S. mansoni* (13,197 sequences) [26] and UniGene data from NCBI for *S. japonicum* (10,809 sequences). Putative transcriptome data were also predicted in-house (15,685 sequences) [27]. Redundant sequences were eliminated according to a coverage ≥90% and identity ≥80% using cd-hit-v4.3 software (http://bioinformatics.ljcrf.edu/cd-hi/). A total of 21,861 target sequences (20,194 sequences derived from *S. japonicum* and 1,667 sequences derived from *S. mansoni*) were determined, and 131,092 experimental probes with 3 or 4 unique 60-mer oligonucleotide probes per sequence corresponding to both strands of the genomic DNA were designed. The microarray was manufactured by Roche NimbleGen (Basel, Switzerland), and microarray hybridisation was carried out at the core facility of the Capital BioCorporation (Beijing, China). The procedure for microarray design and hybridisation was performed as described previously [28]. An expression dataset of target genes was generated based on the fluorescence intensity output from the Roche NimbleGen platform. The expression data of the genes encoding nucleases were retrieved and deeply analysed using Microsoft Excel and GraphPad Prism 5, and heat maps were generated using ClustlX 3.0 and Treeview. The expression levels of the genes at each developmental stage (including the cercariae, schistosomula, male adult, and female adult) were obtained by comparing them with those at the egg stage. Expression levels ± ≥ 2-fold (p < 0.05) were regarded as significantly up-regulated. The microarray data were further validated by two independent QPCR assays [28]. The expression levels of six putative DNase genes (AAW25685.1, Sjp_0035270, AAW25360.1, AAW26473.1, CAX69654.1, and CAX72944.1) were also analysed in this study (data not shown). Quantification of the expression of each gene was performed by normalisation against the expression of the housekeeping gene, NADPH. Statistical analyses were conducted using GraphPad Prism 5 or Microsoft Excel.

### Prediction of nucleases encoded in *S. japonicum* genome

For the prediction of potential *S. Japonicum* nucleases, nuclease protein sequences of 7 species (*Brugia malayi*, *Caenorhabditis briggsae*, *Caenorhabditis elegans*, *Hydra vulgaris*, *Nematostella vectensis*, *Schistosoma mansoni*, and *Trichoplax adhaerens*) were selected from the KEGG database. These 7 species are the most closely related to flatworms according to the KEGG Organisms categories (http://www.genome.jp/kegg/catalog/org_list.html). We constructed an *S. japonicum* protein database including information from the following three sources: *S. japonicum* proteins from NCBI (25319 proteins), *S. japonicum* proteins from CHGC (13469 proteins) and proteins predicted from the 21861 genes described above by Augustus software. Protein sequences of *S. japonicum* were compared with reference nuclease sequences using BLASTP with an E-value < 1e-9. Potential nuclease sequences of *S. japonicum* were further analysed for conserved motifs and domains using the Conserved Domain Database (CDD) v3.08 of NCBI [29,30]. The CDD content includes NCBI-curated domains, as well as domain models imported from a number of external source databases, including Pfamv.22.0 [31,32], SMART v.5.0 [33], COG [34], PRK [35], and TIGRFAM v.13 [36]. Additionally, proteins that were shorter than 100 residues or with lengths of less than 80% of the nuclease core sequences were not retained, and the remaining sequences were subjected to further analysis.

### Sequence comparison and phylogenetic analysis of Sjda with homologous genes from other species

The homologous sequences of *S. japonicum* AAW25360.1 encoding a putative DNase (Sjda) were retrieved from NCBI by performing a BLAST search (version 2.2.26) based on bidirectional best-hit (BBH) identification, an E-value <1e-10 and an identity >30%. Ten sequences from animals, parasites and humans were obtained and subjected to phylogenetic analysis. Multiple alignments of the selected sequences were performed with DNAMAN for sequence comparison or with MUSCLE algorithm implemented in CLC Sequence DNA Workbench 6.6.2 software (CLC bio). A phylogenetic tree was constructed using the maximum likelihood approach implemented in PhyML3.0 [37]. Bootstrap values, expressed as a percentage of 100 replicates, were given at branching points.

### Plasmid construction and recombinant protein generation

Recombinant Sjda-GST and GST proteins of *S. japonicum* were generated for confirmation of the DNase activity of Sjda. A recombinant Sjda-His protein was generated to detect antibodies in the mouse sera. Briefly, the gene fragment encoding Sjda was amplified from *S. japonicum* cDNA using a high-fidelity Phusion DNA polymerase (FinnzymesOy, Finland). PCR was performed with the following primers, which carried an EcoR I and SalI restriction site, respectively: forward 5′-GAG GAA TTC ATG CTC GTA TTT CTG GGA C-3′ and reverse 5′-CTT GTC GAC TTA GGC ATA CAT AAT AAA AAC ATA TTG-3′. The amplified product was purified using a DNA Gel Extraction Kit (Axygen, CA, USA) and cloned into pGEX-4 T-1 and pET-32a expression vectors. The recombinant plasmids were transformed into DH5α (DE3) *Escherichia coli,* and positive clones were selected for sequencing. The correct recombinant plasmids were then transformed into Rosetta *E. coli* for protein expression. The recombinant proteins Sjda-GST and GST were purified with Glutathione Sepharose™ (GE Healthcare, Uppsala, Sweden), and Sjda-His was purified with Ni-NTA Agarose (QIAGEN) according to the manufacturer’s instructions. All proteins were analysed with a 12% SDS-PAGE gel and Western blotting with monoclonal antibodies against the His-tag or GST-tag (all from Cell Signaling Technology, MA, USA).

### Detection of DNase activity of Sjda

Genomic DNA from humans, rabbits, mice, and calves, bacterial plasmid DNA (pGEX-6p-1) and Xhol-digested linear plasmid DNA were used as substrates for the assessment of DNase activity. Human whole blood, New Zealand white rabbit ear tissue and C57BL/6 mouse tail tissue were used for the extraction of genomic DNA with a TIANamp Genomic DNA Kit (Tiangen Biotech., Beijing, China) according to the manufacturer’s instructions. Calf genomic DNA was purchased from Sigma Aldrich (St. Louis, MO, USA). The recombinant Sjda protein and 200 ng genomic DNA were mixed in 25 μl PBS with different pH values, prepared on ice and incubated at 37°C from 5 min to 1 h. The reactions were terminated by incubation at 70°C for 20 min. A 10-μl aliquot of each reaction mixture was analysed by 0.6% agarose gel electrophoresis.

### Detection of Sjda protein in *S. japonicum* parasites

To confirm the expression of the Sjda protein in the parasites, a monoclonal antibody to Sjda was generated. Briefly, the amino acid sequence of Sjda was analysed for antigenic determinants, hydrophilicity and secondary structures using Geneious, and a 237-bp gene fragment (from amino acid 25 to 108) was chosen and cloned into pET-30a plasmids to generate a recombinant protein. The purified recombinant protein was used to immunise BABL/c mice (females, 6 weeks old). Splenocytes from the immunised mice were fused into SP2/O cells. The positive fusion cells were selected to generate a monoclonal antibody against Sjda. The specificity of this antibody was proven by Western blotting.

Parasites at all stages stored at −80°C were homogenised by grinding in liquidnitrogen followed by incubation with lysis buffer (8 M urea, 4% CHAPs, 1% DTT, 1% EDTA, 10 mM Tris, and 35 μg/ml PMSF) for 30 min on ice. The mixture was centrifuged at 12,000 rpm for 30 min at 4°C, and the protein concentrations were quantified with a BCA kit (Pierce, Rockford, IL, USA) in accordance with the manufacturer’s directions. The extracted proteins were denatured by boiling in SDS-PAGE buffer, separated on 12% SDS-PAGE gels, and transferred to polyvinylidenedifluoride membranes (Millipore, Bedford, MA, USA). The blots were blocked with a blocking buffer containing 5% skim milk for 1 h at room temperature and incubated in the same buffer with an anti-Sjda monoclonal antibody (2 mg/ml, 1:1,000 dilution) or mouse IgG control overnight at 4°C. After washing, detection was accomplished by incubation with an IRDye 800 CW conjugated goat anti-mouse IgG (H + L) antibody (Li-COR Biosciences, Lincoln, NE, USA), using Odyssey (Li-COR).

Localisation of the Sjda protein in adult worms was carried out with an immunofluorescence assay using anti-Sjda monoclonal antibodies according to the standard protocol with minor modifications. Briefly, parasites were fixed in 4% formaldehyde and then permeabilised for 2 h with 1% SDS in PBS followed by treatment with proteinase K (2 μg/ml) for 10 min at room temperature. After permeabilisation, the parasites were re-fixed for 10 min in 4% formaldehyde and rinsed with PBSTx (PBS plus 0.3% Triton X-100). The parasites were incubated in blocking solution (5% horse serum, 0.3% Triton X-100, and 0.05% Tween 20 in PBS) for 2 h at room temperature. An anti-Sjda monoclonal antibody (2 mg/ml) diluted 1:500 in blocking solution was incubated with the parasites at 4°C overnight. Parasites incubated with normal mouse IgG (Calbiochem, Darmstadt, Germany) were included as controls. After being washed four times (for 30 min each), the parasites were further incubated with Alexa Flour 555 donkey anti-mouse IgG (H + L) and DAPI (all from Invitrogen, OR, USA) at 4°C overnight. Fluorescence was visualised with a TCS SP5 confocal microscope (Leica Microsystems, Wetzlar, Germany).

### Detection of anti-Sjda antibodies in host sera by Western blotting

Mouse sera were collected on days 0, 7, 14, 28 and 42 post-infection and stored at −80°C. Recombinant Sjda-His was resolved in 12% SDS-PAGE gels and transferred to polyvinylidenedifluoride membranes. Sera from infected mice were used as primary antibodies (diluted 1:200), and sera from uninfected mice were used as a control. Anti-Sjda antibodies in the mouse sera were detected by Western blotting as described above.

## Additional files

Additional file 1:
**Dynamics of Sjda-specific antibodies in host sera.** A. Sjda-specific antibodies in the sera of *S. japonicum*-infected mice on0, 7, 14, 28 and 42 days post-infection were detected by Western blotting. Sera collected at each time point were pooled. Lane M, protein molecular weight markers. B. Percentages of sera with specific anti-Sjda antibodies at the indicated time points post-infection detected by Western blotting. Three independent experiments were carried out, with 5–7 mice in each group. Additional file 2:
**WB images of the recognition of the Sjda-His protein by antibodies in each** ***S. japonicum-*** **infected mouse at the indicated infection time points.**
Additional file 3:
**Characteristics of putative nuclease sequences of** ***S. japonicum.***
