# Supplementary figures and images for: A novel Schistosoma japonicum endonuclease homologous to DNase II
Source: BMC Genomics. 2015 Feb 25;16(1):126. doi: 10.1186/s12864-015-1319-5 (PMC4349608; doi:10.1186/s12864-015-1319-5)

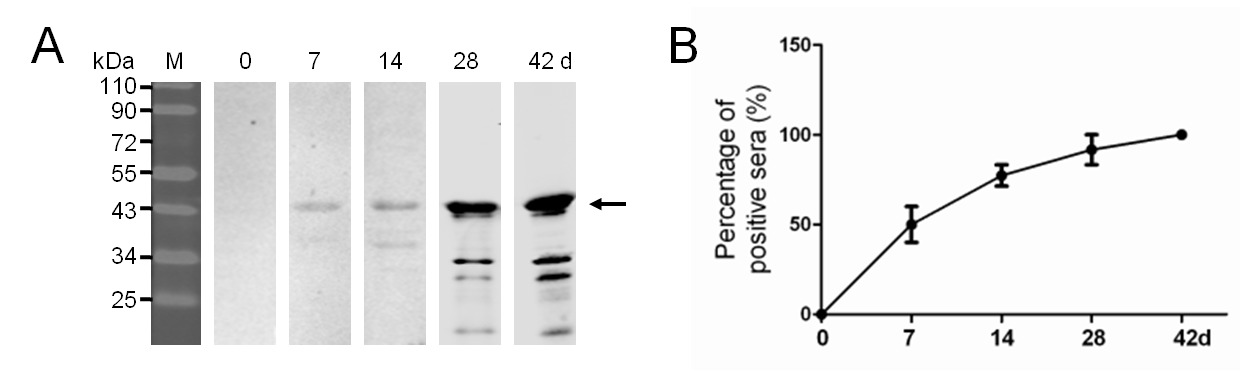

Supplement: Additional file 1: — Dynamics of Sjda-specific antibodies in host sera. A. Sjda-specific antibodies in the sera of S. japonicum-infected mice on0, 7, 14, 28 and 42 days post-infection were detected by Western blotting. Sera collected at each time point were pooled. Lane M, protein molecular weight markers. B. Percentages of sera with specific anti-Sjda antibodies at the indicated time points post-infection detected by Western blotting. Three independent experiments were carried out, with 5–7 mice in each group. [file 12864_2015_1319_MOESM1_ESM.tiff]

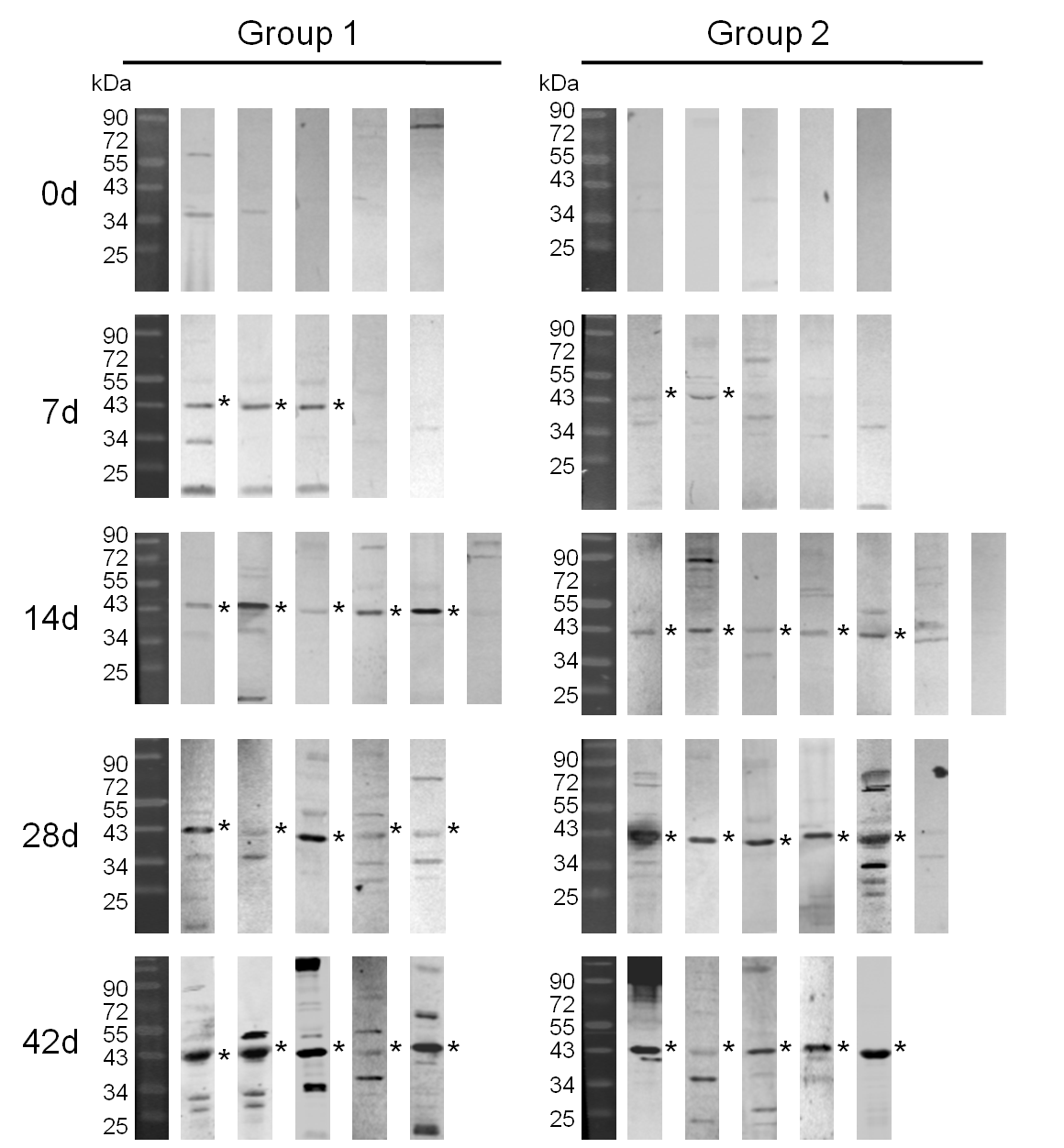

Supplement: Additional file 2: — WB images of the recognition of the Sjda-His protein by antibodies in each S. japonicum- infected mouse at the indicated infection time points. [file 12864_2015_1319_MOESM2_ESM.tiff]
